# Modeling the Encephalopathy of Prematurity in Animals: The Important Role of Translational Research

**DOI:** 10.1155/2012/295389

**Published:** 2012-05-23

**Authors:** Hannah C. Kinney, Joseph J. Volpe

**Affiliations:** ^1^Department of Pathology, Children's Hospital Boston and Harvard Medical School, Boston, MA 02115, USA; ^2^Department of Neurology, Children's Hospital Boston and Harvard Medical School, Boston, MA 02115, USA

## Abstract

Translational research in preterm brain injury depends upon the delineation of the human neuropathology in order that animal models faithfully reiterate it, thereby ensuring direct relevance to the human condition. The major substrate of human preterm brain injury is the encephalopathy of prematurity that is characterized by gray and white matter lesions reflecting combined acquired insults, altered developmental trajectories, and reparative phenomena. Here we highlight the key features of human preterm brain development and the encephalopathy of prematurity that are critical for modeling in animals. The complete mimicry of the complex human neuropathology is difficult in animal models. Many models focus upon mechanisms related to a specific feature, for example, loss of premyelinating oligodendrocytes in the cerebral white matter. Nevertheless, animal models that simultaneously address oligodendrocyte, neuronal, and axonal injury carry the potential to decipher shared mechanisms and synergistic treatments to ameliorate the global consequences of the encephalopathy of prematurity.

## 1. Introduction

Translational research in the brain injury of premature infants involves the delineation of basic mechanisms and therapeutic strategies in animal models and their subsequent transformation into human clinical trials to improve neurological outcome. Yet, from the outset, advances in our understanding of preterm brain injury are directly contingent upon neuropathologic studies in humans. Indeed, translational research depends upon the initial delineation of the basic neuropathology in the human brain and then development of animal models that faithfully reiterate this pathology, thereby ensuring direct relevance to the human condition. The major neuropathologic substrate of human preterm brain injury is the encephalopathy of prematurity (EP), a term coined to characterize the multifaceted gray and white matter lesions in the preterm brain that reflect acquired insults, altered developmental trajectories, and reparative phenomena in various combinations [1–4]. The encephalopathy of prematurity also is associated with hemorrhages, notably in the germinal matrix of the ganglionic eminence and cerebellum and with focal micro or macroinfarcts [5–7]. Because EP occurs at a time of rapid brain growth, the insult may impact a host of developmental programs, resulting in maturational defects that compound the acquired lesion, for example, hypoxic-ischemic injury leading to loss of pre-OLs in turn leading to impaired myelination. The cause of EP is multifactorial, and includes cerebral hypoxia ischemia and systemic infection/inflammation that results in glutamate, free radical, and/or cytokine toxicity to pre-OLs, axons, and neurons [8]. In addition, other maturation-dependent biochemical derangements likely contribute to EP caused by the multiple extrauterine insutls that the preterm infant experiences and is not developmentally equipped to defend against [3]. One example is bilirubin toxicity that may contribute to the (nonspecific) neuronal loss and gliosis seen in the basal ganglia in EP [3, 7, 9]. Given the heterogeneity and diverse combinations of the lesions that comprise EP, it is not surprising that the spectrum of neurodevelopmental abnormalities in preterm survivors is wide and includes, often in combination, deficits in executive functions [10, 11], autistic behaviors [12], cerebral palsy [13], and visual cognitive impairments [14].

The goal of the following review is to highlight key features of EP that we believe are critical to model in animal paradigms. The patterns and mechanisms of injury in EP are highly dependent upon the specific maturational stages of OLs, neurons, and axons over the last half of gestation, that is, the time frame of EP. We begin with a brief overview of events in human preterm brain development that are particularly relevant to animal modeling (Table 1), given that the vulnerability of pre-OLs, axons, and neurons to injury in EP is critically dependent upon specific maturational stages. We then define the major components of the neuropathology of EP that animal models need to consider (Table 2). We conclude with a consideration of the interplay between human and animal analyses in translational research and the need for the two types of analysis to inform and build upon each other towards the complete elucidation of EP and its treatment.

## 2. The Development of the Brain in the Last Half of Human Gestation

The encephalopathy of prematurity spans the last half of human gestation, a spectacular and complex period in brain growth and development. From midgestation to term, the brain weight increases dramatically by 90%, and the cerebral cortex changes from a smooth surface with only the Sylvain fissure to a complex pattern with all primary, secondary, and tertiary gyri [7, 15]. The ganglionic eminence is thickest at 20–26 gestational weeks and involutes by 34–36 weeks; until 18 weeks, proliferating cells as identified by Ki67 immunoreactivity are present throughout the ganglionic eminence, after which they persist ventrally until about 28 weeks and then markedly decrease [5]. Among the many interrelated developmental events that occur during this critical period of brain growth, several are particularly germane to modeling EP in animals because they relate to cellular vulnerabilities to glutamate, free radical, and cytokine toxicities, as illustrated in the cerebral white matter and cortex (Table 1).

### 2.1. Vascular Development in the Cerebral White Matter in the Preterm Period

Immature vascular end zones irrigate distal fields in the preterm cerebral white matter [3, 8, 16–18], and thus the deep and periventricular regions are susceptible to very low basal blood flow, documented in human preterm brain by multiple techniques [8, 17, 19, 20]. This vascular immaturity is characterized by decreased numbers of vascular perforators from the leptomeninges in the white matter compared to the cortex [16]. Moreover, the intrinsic capillary plexus of the white matter has fewer and longer capillaries and larger intercapillary spaces than that of the cortex [16]. Functional cerebral vascular autoregulation is also underdeveloped in the premature infant, with a propensity for a pressure-passive cerebral circulation [8, 17, 19, 20]. Thus, the “margin of safety” for blood flow of the deep and periventricular cerebral white matter is compromised due to its developmental anatomic and physiological features, and these regions are vulnerable to fluctuations in blood pressure, as well as overt hypotension, common complications of pulmonary immaturity, respiratory distress syndrome, and accompanying mechanical ventilation in premature infants.

### 2.2. Oligodendrocyte (OL) Development in the Cerebral White Matter in the Preterm Period

The peak window of vulnerability to PVL, that is, 24–36 weeks, coincides with the period of dominance of pre-OLs [21, 22]. Oligodendrocyte maturation from an OL progenitor into a myelinating OL involves a sequence of developmental stages, each characterized by a progressively complex morphology and the expression of stage-specific markers. This progression includes the following OL-developmental stages in increasing order of maturation as defined by cell-specific antibodies: (1) A2B5-expressing OL progenitors; (2) O4-expressing precursor OLs; (3) O1-expressing immature OLs; (4) myelin basic protein (MBP-) expressing mature OL [21–24]. Neural stem cells give rise to OL precursor cells around 13 gestational weeks that proliferate and migrate throughout the brain, and then differentiate into pre-OLs around 20 weeks [23, 24]. The O4-positive cell comprises approximately 90% of the total OL population in the preterm brain until about 28 gestational weeks and accounts for at least 50% of the total population until term [21]. In contrast, the O1-positive OL population accounts for only about 10% of the total OL population in the preterm infant and does not approach 50% of the total OL population at term [21]. OLs producing MBP first appear around 30 gestational weeks in the cerebral white matter [21], but active myelin sheath production, as detected by Luxol-fast-blue which stains myelin sheath phospholipids, does not begin until 3 postnatal months [25]. Once an axon makes synaptic contact with its target cell, wrapping of the axon by the myelin sheath begins, a process which depends upon both axonal and OL maturation and multiple signaling factors between them, many of which are yet unknown [26]. In the human cerebral white matter, OL contact with the axon involves the extension of immature, O4+/O1+/MBP-, “pioneer” processes that extend longitudinally along the length of the axon [22]. Once axonal contact is made and myelination initiated, OLs transport myelin proteins and lipids to the myelin membrane [26].

### 2.3. The Development of Glutamate Receptors in Cerebral White Matter in the Preterm Period

Given the critical role of glutamate receptors in mediating excitotoxicity, their regional and cellular development is of major interest in the preterm brain. Several studies of glutamate receptor subtypes have been performed in perinatal human brains [27–29], including a comprehensive cellular localization of alpha-amino-3-hydroxy-5-methyl-4-isoxazole-propionic acid (AMPA) receptor subtypes in the human cerebral cortex and white matter from the preterm period into infancy [29]. Alpha-amino-3-hydroxy-5-methyl-4-isoxazole-propioinic acid receptor subtypes lacking GluR2 are especially permeable to calcium and therefore likely to convey increased susceptibility to hypoxia ischemia. From 20 to 37 weeks, pre-OLs, radial glial fibers (RGFs), and subplate neurons express AMPA receptors that lack GLuR2 expression [29]. The glutamate transporter EAAT2, on the other hand, is transiently expressed in the cerebral white matter in the period of vulnerability to PVL and likely increases the susceptibility of this site to excitotoxicity because it is a major source of extracellular glutamate complicating ischemic injury [30]. This transporter is expressed by OLs but not astrocytes or axons in the preterm white matter [30].

### 2.4. Development of Microglia in the Cerebral White Matter in the Preterm Period

Microglia are abundant in the human forebrain around 16–22 gestational weeks [31–33]. They are involved in a variety of normal developmental events, such as axonal development, angiogenesis, and synaptic pruning [33]. Their density in the cerebral white matter reaches a peak around the early third trimester and then declines rapidly after 37 gestational weeks [33]. As the microglial density declines in the white matter, it increases in the cortex, perhaps reflecting continuing migration of microglia [33]. Thus, microglia are transiently elevated in the peak window of vulnerability to PVL, well situated to become activated and lead to free radical and cytokine injury to pre-OLs [8]. Of note, O4 OLs in the human cerebral white matter express interferon-*γ* receptors, indicating that they are vulnerable to receptor-mediated mechanisms by cytokines [34].

### 2.5. Development of Radial Glial Fibers and Astrocytes in the Cerebral White Matter in the Preterm Period

The last half of human gestation is a crucial time in astrocyte formation in the cerebral cortex and white matter. The radial glial cell originates in the ventricular/subventricular zone and retains connections with the ependyma and pia [16]; it is capable of generating neurons and astrocytes [35]. Its long, thin, and linear processes, that is, RGFs, serve as a guide for migrating neuroblasts and glial cells [16]. Glutamatergic neurons form in the dorsal telencephalic pallium and migrate along RGFs early in gestation [36]. In the human brain, in contrast to the rodent brain, approximately two-thirds of GABAergic neurons arise from the dorsal telencephalic zone and migrate along RGFs; the remaining one-third originates in the ganglionic eminence and migrates tangentially to the cortex [37]. From 19 to 30 weeks, RGFs are abundant; around 30-31 weeks, they begin to transform into fibrous astrocytes in the white matter and from 30 weeks to term gestation (37–41 weeks), they progressively disappear as the white matter becomes increasingly populated with transformed astrocytes [38–40]. By term, RGFs completely disappear, thereby definitively marking the end of radial migration. Fibrous astrocytes in the white matter also form from glial precursors that migrate outward from the ventricular/subventricular zone independent of RGFs [16]. Reactive gliosis with gemistocytic morphology and GFAP-positive immunostaining begins around midgestation in the human brain [7].

### 2.6. The Development of Antioxidant Systems in the Cerebral White Matter in the Preterm Period

The human preterm brain is susceptible to free radical injury in hypoxia ischemia because of a relative developmental deficiency in antioxidant enzymes. These enzymes include the superoxide dismutases-1 and -2 for the conversion of superoxide anion oxygen to hydrogen peroxide and catalase and glutathione peroxidase for the breakdown of hydrogen peroxidase. In the cerebral white matter of the preterm infant, the expression of both superoxide dismutased significantly lags behind that of catalase and glutathione peroxidase [41]. These enzymes are all expressed by OLs and astrocytes in the preterm brain and exceed adult levels at 2–5 postnatal months, the peak period of active myelin sheath synthesis in cerebral white matter [41]. Indeed, human myelination with the rapid production of cellular membrane is associated with the “physiological” generation of free radicals resulting in lipid peroxidation [42].

### 2.7. Axonal Development in the Cerebral White Matter in the Preterm Period

The second half of human gestation is characterized by axonal elongation from the early migrating pyramidal neurons, as well as from cortical afferents from the thalamus and other cortical and subcortical structures [43]. Central axons elongate, search out their proper targets, and establish synaptic contacts; initial axonal excess is followed by axonal elimination or pruning, leading to refinements in connectivity [26]. The expression levels of growth-associated protein-43 (GAP-43), a neuronal membrane phosphoprotein and marker of axonal elongation, are low in the human cerebral white matter at 19-20 weeks, increase approximately 3-fold within two weeks, peak at term at approximately 5-times adult levels, and decrease dramatically at approximately 17 postnatal months, with adult levels attained in the second year [44]. From 14 gestational weeks onward, so-called “crossroads” of intersecting projection and associative axons are present in the periventricular zones, directly adjacent to the lateral ventricles, coinciding with the sites of predilection for focal necrosis in PVL [45]. Because of the complexity of the axonal pathways in these discrete and restricted loci, discrete lesions within them are postulated to result in diverse and multiple functional impairments simultaneously in preterm survivors [44].

### 2.8. Neuronal Development of the Cerebral Cortex in the Preterm Period

During the last half of gestation, the neocortex transforms from an undifferentiated cortical plate to a highly specialized structure [7, 16, 46–48]. Around 30 gestational weeks, the cortical plate becomes comprised of six layers in which each layer is characterized by a specific composite of differentiating pyramidal and nonpyramidal neurons [46]. The cortex increases in thickness due to striking increases in the neuropil, for example, neuronal cell size, dendritic arborization, spine formation, and arrival of preterminal afferents [7, 16, 46–48]. Indeed, the number of specific gene expression and alternative splicing patterns are large and associated with distinct regions and developmental processes [49]. Relative to excitotoxicity, GluR2 is low in the pyramidal and nonpyramidal neurons in the cerebral cortex during the term neonatal period [29].

### 2.9. Late Development of the GABAergic System in the Cerebral Cortex in the Preterm Period

A defining feature of cortical development in the human preterm period is the late development of the GABAergic interneurons that play a key role in cortical specification, output, and synaptic plasticity [16, 36, 50, 51]. At least 20% of GABAergic neurons migrates through the white matter to the cerebral cortex over late gestation [50]. This migration peaks around term and then declines and ends within the first 6 postnatal months; in parallel, the GABAergic neuronal density increases in the cortex over late gestation, peaks at term, and declines thereafter [50]. From midgestation to infancy, the pattern of GABA_A_ receptor binding also changes from uniformly low across all cortical layers to high levels concentrated in the middle laminae [50]. This developmental profile may reflect the ingrowth of glutamatergic thalamocortical fibers during this time period with a parallel upregulation of inhibitory (GABAergic) modulation in the middle laminae to counterbalance the increase in excitatory inputs during the preterm period. The GABA_A_ receptors are likely excitatory because of relatively high intracellular levels of chloride. The latter occurs because of a developmental imbalance of the chloride importer (NKCC1) and the chloride exporter (KCC1). In the human cerebral cortex, the expression of NKCC1, causing chloride influx, rapidly increases from 20 gestational weeks to term and then dramatically decreases, reaching a plateau after 4 postnatal months [52]. The expression of KCC2, causing chloride influx, on the other hand, is low during the preterm period but increases dramatically postnatally to adult levels [52]. Thus, in the preterm period, GABA agonists lead to chloride efflux and depolarization/excitation, rather than the normal chloride influx and hyperpolarization/inhibition.

### 2.10. Development of Protoplasmic Astrocytes in the Cerebral Cortex in the Preterm Period

 In the cerebral cortex, astrocyte precursors that have migrated upward along the RGFs to Layer I, differentiate and send processes towards the developing cortical blood vessels, and gradually transform into the protoplasmic astrocytes of the cortex [16]. This event occurs “late”, that is, after the completion of the migration of neuroblasts destined to form cortical neurons. These astrocytes express the glutamate transporters EAAT1 and EAAT2 but not glial acidic protein (GFAP) in the late preterm period [53]. Discrete EAAT1/EAAT2 astrocytic “patches” appear in the developing cortex in the late preterm period [53]. The patches may reflect nonoverlapping astrocytic territories that potentially contribute to the synchronization of neurons [53].

### 2.11. Development of the Subplate Region in the Preterm Period

Subplate neurons are among the first generated neurons of the neocortex and come to lie immediately beneath the developing cortical plate where they form part of the early neocortical circuitry [54, 55]. During early development, subplate neurons play a critical role in the establishment of connections between the cortex and thalamus and cortical lamination [54, 55]. These neurons are also critical in the establishment of axons destined to the cortex from the contralateral cerebrum (commissural-cortical fibers) and from unilateral cortical sites (corticocortical association fibers). There is no available immunomarker that specifically labels human subplate neurons in tissue sections. Their phenotype is defined by morphology, location, and connectivity with the cortical plate. In the human subplate region, fusiform, granular, unipolar, bipolar, multipolar, and inverted pyramidal neurons have been observed [54, 56]. The specific function of each morphological subtype is unknown. Glutamatergic, GABAergic, and other transmitter-specific neurons, transporters, and receptors have also been observed in the human subplate region [29, 50, 51, 56]. Kostovic and Rakic divide the developmental stages of the human subplate zone into the presubplate stage at around 12-13 gestational weeks, the subplate formation stage around 12–15 weeks, the subplate stage around 15–35 weeks, and the subplate dissolution stage beyond 35 weeks [54]; the latter two stages coincide with the period of EP. In the subplate stage (15–35 weeks), the subplate serves as a “waiting” compartment for the competition, segregation, and growth of afferents originating from the thalamus, brainstem, basal forebrain, and ipsi- and contralateral cerebral hemisphere [43, 54]. During this stage, the histochemical stain acetylcholinesterase highlights cholinergic terminals arising from the basal forebrain [54]; around midgestation, this stain indicates that the subplate zone is four times thicker than the cortical plate [54]. After the incoming fibers enter the cortical plate around 32 weeks, the subplate zone almost completely disappears, leaving only a “vestige” of neurons cells scattered throughout the subcortical white matter, that is, so-called interstitial neurons, which nevertheless contribute to the modulation of adult cortical processing [57].

### 2.12. The Development of Subcortical Structures in the Preterm Period

The involvement of the basal ganglia, thalamus, hippocampus, cerebellum, and brainstem in EP suggests that all of these gray matter sites have developmental factors that increase susceptibility to glutamate, free radical, cytokine, and metabolic insults. Nevertheless, information is relatively limited about these factors in the human preterm brain. Transient expression of glutamate receptors has been described in the developing human basal ganglia [28] and brainstem [58, 59]. The expression levels and cellular localization of several antioxidant enzymes have also been defined in the basal ganglia and brainstem by immunohistochemistry [60].

## 3. The Pathology of the Encephalopathy of Prematurity

### 3.1. Periventricular Leukomalacia (PVL)

This lesion is the major white matter component in EP and is defined as focal periventricular necrosis associated with reactive gliosis and microglial activation in the surrounding cerebral white matter [3, 7]. The necrotic foci likely represent a core infarct with destruction of all cellular elements [3, 7, 61], while the astrocytic and microglial response in the surrounding white matter represents the penumbra with less severe and potentially reversible ischemic injury [3]. The necrotic foci progress from coagulative necrosis (characteristic of the histology of tissue ischemia in all tissues [3]), with hypereosinophilia, nuclear pyknosis, and axonal spheroids, followed by organizing necrosis with reactive gliosis, macrophagocytic infiltration and tissue disintegration, and then end-stage cystic formation and gliosis [3, 7]. Importantly, the necrotic foci are not always apparent upon macroscopic examination. In autopsy studies in our hospital from the modern era of intensive care, 46–82% of PVL cases, depending upon the dataset, have only microscopic necrotic foci (with macrophagocytic infiltration) that measure less than 2 mm in diameter [9, 46, 62]. Moreover, neuroimaging studies over the past 10–15 years demonstrate that cystic PVL has declined in incidence and noncystic PVL has become the dominant lesion, accounting for more than 90% of PVL and occurring in approximately 50% of very low birth weight infants [2]. Nevertheless, visually obvious foci of necrosis, so-called “white spots”, as well as cysts greater than 2 millimeter in diameter are still detected at autopsy [9, 46, 62]. Diffuse white matter gliosis without periventricular necrotic foci occurs in preterm brains [63] but its relationship to PVL is uncertain, for example, whether or not it represents the least severe end of a spectrum of ischemic injury to the premyelinated white matter, with PVL at the most severe end. Nevertheless, neuronal loss in gray matter sites occurs almost exclusively in association with PVL and not with diffuse white matter gliosis [9], suggesting that PVL is the hallmark of EP and that injury that leads to focal necrosis in the white matter *and* neuronal loss in the gray matter is the defining event of EP.

The pathogenesis of PVL involves acute loss of pre-OLs [64, 65]; some OL cell bodies appear to survive with loss of cell processes [66], others with morphological dysfunction in myelin formation [66], as well as hypomyelination [67]. Immunocytochemical analysis using an antibody to Olig2, a pan-OL lineage marker, indicates no significant difference in Olig2 cell density in the periventricular or intragyral white matter between PVL cases and controls [66]. Nevertheless, early lineage markers are needed to determine if there is arrested OL maturation with dominance of pre-OLs over mature OLs. Qualitative abnormalities of MBP staining in both the diffuse and necrotic foci of PVL occur despite preserved OLIG2 cell density [66]. They include excessive MBP immunostaining in enlarged OL perikarya that presumably reflects a functional derangement in MBP transport from its site of production in the OL cell body to the OL processes [66]. Free radical injury to pre-OLs in PVL is indicated by immunocytochemical evidence for protein nitration and lipid peroxidation of pre-OLs in the diffusely gliotic component of PVL [64]. In addition, F(2)-isoprostanes, an arachidonate metabolite/lipid peroxidation marker of oxidative damage, is significantly increased in the white matter of early PVL cases [65]. The end-stage of PVL is delayed or hypomyelination of the cerebral white matter and compensatory ventricular enlargement [7].

### 3.2. Gray Matter Lesions in EP

Neuronal loss and/or gliosis are the histopathologic hallmarks of gray matter injury in EP and occur in virtually all gray matter sites, albeit in variable combinations [9]. Over one-third of PVL cases demonstrates gray matter lesions characterized by neuronal loss and/or gliosis [9]; microglial activation is oftentimes striking. Of note, more refined techniques, such as analysis of dendritic and spine number and morphology, may ultimately detect neuronal deficits at the subcellular (and molecular) levels. The incidence of neuronal loss, as assessed semiquantitatively in tissue sections, is 38% in the thalamus, 33% in the globus pallidus and hippocampus, and 29% in the cerebellar dentate nucleus [9]. Gliosis without obvious neuronal loss is more common than combined neuronal loss and gliosis, occurring in the thalamus (56% of PVL cases), globus pallidus (60%), hippocampus (47%), basis pontis (100%), inferior olive (92%), and brainstem tegmentum (43%) [9]. In a histopathologic survey of brain injury in very low birth weight infants, the frequency of neuronal loss (sites unspecified) is reportedly less than cerebral white matter abnormalities [68]. The basis of neuronal injury in PVL may be heterogeneous, as suggested in the thalamus [69]. At this site, injury occurs in four different patterns, that is, diffuse gliosis with or without neuronal loss, microinfarcts with focal neuronal loss, macroinfarcts in the distribution of the posterior cerebral artery, and status marmaoratous [69]. These different patterns likely each reflect separate mechanisms, including diffuse hypoxia ischemia and focal arterial embolism [69], as well as potential different temporal characteristics of the responsible insults.

The cerebellum in the preterm infant demonstrates bilateral, symmetric deficits in hemispheric volume without overt parenchymal hemorrhage or infarction [60–72]. This reduced volume is commonly associated with intraventricular or subarachnoid hemorrhage [72]. Moreover, cerebellar underdevelopment is associated with supratentorial lesions, especially PVL and posthemorrhagic infarction, suggesting the possibility of transsynaptic mechanisms in its pathogenesis via corticopontocerebellar pathways [70, 72]. Yet, neuroimaging studies also indicate a gradual deficit in cerebellar volume in preterm infants associated with infratentorial hemosiderin deposition in the majority of cases [72]. Thus, it has been postulated that blood products (hemosiderin/nonheme iron) in the cerebrospinal fluid lead to cerebellar underdevelopment due to their toxic effects upon the proliferating granule precursor cells of the external granular layer which are located directly at the interface with the subarachnoid space and which migrate inward to form the internal granular layer [72]. Nevertheless, this idea, based upon neuroimaging studies, has not been verified by quantitative analysis of the cell number of the internal granular layer. Indeed, semiquantitative analysis of the cerebellum has revealed moderate loss of cortical neurons in 24% of cases with PVL and moderate loss of dentate neurons in 29% of cases [9] in association with reactive astrocytes. Thus, the neuropathologic features (notably gliosis) suggest an acquired insult leading to cerebellar atrophy rather than underdevelopment as the basis of the small size of the cerebellum on neuroimaging studies. Yet, the distinction between atrophy and underdevelopment is difficult in the developing cerebellum in which migration from the external to the internal granular layer is protracted over the last half of gestation into infancy. That is, a particular insult may simultaneously lead to *atrophy* with drop out of cells already at their proper address (inciting gliosis) *and underdevelopment* due to disruption of still migrating cells and an incomplete complement of neurons. Indeed, the pathology of the cerebellum epitomizes the so-called “complex amalgam” of EP where developmental and destructive processes intersect [2]. In regards to the cerebellar relay nuclei, it is uncertain if the neuronal loss in the basis pontis and inferior olive, the major cerebellar relay nuclei, which is seen in 21% of PVL cases [9], is primary or secondary to transsynaptic degeneration.

### 3.3. Deficit of Neurons in the Subplate Zone and White Matter in EP

Not only is there damage to neurons in gray matter sites but also to neurons located in the white matter and subplate region. The density of granular neurons is significantly reduced in the periventricular and central white matter and subplate region in PVL [56]. These neurons are likely late migrating GABAergic neurons and/or non-GABAergic constituents of the subplate region and interstitial white matter [56]. In regard to the former possibility, a reduction in the density of GAD67-immunopositive neurons and neurons expressing the GABA_A_ *α*1 receptor has been reported in human perinatal white matter lesions (with and without focal necrosis) [51]. The granular neurons expressed GAD67/65, a marker of the GABAergic phenotype, but not markers of neuronal and glial immaturity (Tuj1, doublecortin [DCX], or NG2) [56]. Notably, in contrast to granular neurons, there is not a consistent deficit in unipolar, bipolar, multipolar, or inverted pyramidal neurons in the white matter or subplate region in PVL [56]. The finding of reduced density of white matter neurons in the necrotic foci in PVL is not unexpected since necrosis involves destruction of all cellular elements. The deficit in the granular neurons distant from the focally necrotic lesions, that is, in the subplate region, on the other hand, is of major interest because it occurs presumably in zones of less severe insult. The preferential damage to granular neurons, including distant from the necrotic foci, suggests that this particular subtype is exquisitely sensitive to hypoxia ischemia. In humans, approximately one-third of GABAergic neurons arises from the ganglionic eminence [37]. In the study of reduced granular cells in the white matter and subplate region, approximately one-third of the PVL cases had germinal matrix hemorrhages in the ganglionic eminence, raising the possibility that the reduction in neuronal density in the white matter in these PVL cases was accentuated by mechanical damage to the GABAergic neurons originating in this site. It has recently been reported that germinal matrix hemorrhage is associated with a marked decrease in proliferating cells, as identified by Ki67 immunoreactivity and not an increase in apoptosis, in survivors over 12 hours [5]. Yet, there was no significant difference in the granular neuronal density in the white matter between PVL cases with and without ganglionic hemorrhages.

### 3.4. Damage to RGFs in EP

Radial glial fiber damage could adversely affect radial neuronal migration with secondary maldevelopment of the vertical columns of the cerebral cortex. This idea has not been rigorously tested, however, in the preterm brain with the necessary tissue methods to define quantitative derangements in cortical mini- and macrocolumn formation in postmortem brains. Damage to RGFs may also potentially impair astrocytic development, as fibrous astrocytes in the white matter develop from the transformation of RGFs, and protoplasmic astrocytes in the cortex transform from Layer I astrocytes following RGF migration [16]. A deficit in fibrous and/or protoplasmic astrocytes in EP may be potentially masked by gliosis, as there are no quantitative criteria for an “adequate” astrocytic response. Nevertheless, reactive astrocytes in EP demonstrate evidence of oxidative and nitrative stress, which is potentially primary and could lead to an “inadequate” glial response [64, 73]. Indeed, so-called “acutely damaged glia” in PVL [63] may represent astrocytes undergoing cell death. Given the role of astrocytes in protecting against ischemic injury via glutamate uptake and in orchestrating cytokine responses, damage to them secondary to potential RGF injury in EP potentially is likely to be especially deleterious. The delineation of RGF pathology in EP is an important direction for future research.

### 3.5. Diffuse Axonal Injury in the Cerebral White Matter in EP

With *β*-amyloid precursor protein, axonal spheroids are detected within the necrotic lesions of PVL, whether focal or large [74]. With the apoptotic marker fraction, on the other hand, diffuse axonal injury is detected in the white matter distant from acute or organizing necrotic foci, suggesting a widespread axonopathy in PVL [62]. This diffuse axonal damage may reflect secondary degeneration of thalamocortical afferents complicating primary thalamic neuronal loss. Alternatively, it may be primary due to hypoxic ischemic or inflammatory injury directly to the axon, with secondary impairments in axonal-OL interactions in the initiation and maintenance of myelination. Irrespective of its pathogenesis, widespread axonal damage likely contributes to the reduced white matter volume and callosal thinning in end-stage PVL. Axonal injury throughout the diffuse and focal components of PVL may also lead to architectonic changes in the overlying cerebral cortex [46, 75].

### 3.6. Reactive Gliosis and Activated Microglia in the Cerebral White Matter in EP

Reactive gliosis and activated microglia are the two major inflammatory components of PVL [3, 4, 7]. Presumed to be initially protective against pre-OL cell damage, they carry the potential for compounding tissue injury when the insult is prolonged and/or severe. Reactive gliosis in PVL is preferentially located in the deep as compared to intragyral white matter [64] and thereby defines injury in the vascular distal fields of the cerebral white matter. Activated microglia likewise conform to this regional distribution, while macrophages are prominent in the organizing necrotic foci of the periventricular regions [3, 5, 7]. Both astrocytes and microglia/macrophages produce inflammatory cytokines, and immunocytochemical studies in PVL demonstrate increased cytokine expression within them as a distinctive feature of the histopathology [34, 76]. Notably, reactive astrocytes in PVL express interferon-*γ* and thus are a potential source for this toxic cytokine, particularly to pre-OLs compared to mature OLs [77]. Reactive astrocytes and microglia/macrophages also help protect pre-OLs from excitotoxic injury by the upregulation of the glutamate transporter EAAT and uptake of excessive tissue glutamate, as suggested by the finding that the percentage of EAAT2-immunopositive astrocytes is increased in PVL compared to control white matter, and macrophages in the necrotic foci express EAAT2 [78]. Yet, reactive astrocytes and microglia may contribute to free radical injury in PVL, as indicated by intense expression of inducible nitric oxide synthase (iNOS), a marker of nitrative stress, in reactive astrocytes in the acute through chronic stages of PVL, and in activated microglia primarily in the acute stage, the latter observation suggesting an early role for microglial iNOS in the pathogenesis of PVL [73]. In addition, the density of iNOS-immunopositive cells is significantly increased in the diffuse component [73].

### 3.7. Neural Repair in EP

Evidence is mounting that tissue repair is underway in EP within the neonatal period, that is, within the period of the inciting insult(s). In this regard, Olig2 cell density at the necrotic foci is increased in PVL cases compared with that in sites distant from these foci, suggesting that OLs are migrating to the ischemic core to replenish OL cell number [66]. In PVL, the stem cell immunomarker to nestin demonstrates its increased expression in glia and neurons, attributed to nestin upregulation in response to injury rather than regeneration of new cells [79]. Using DCX immunopositivity as a marker of postmitotic migrating neurons, we found significantly increased densities of DCX-immunopositive cells in PVL cases compared to controls in the subventricular zone, necrotic foci, and subcortical white matter in the perinatal time window, that is, 35–42 postconceptional weeks [80]. These increased DCX-immunopositive neurons may be *en route* to replenish the loss of white matter neurons. Their increased density in the subventricular zone suggests that the regenerative capacity originates in this germinal site [81]. Successful incorporation of the DCX-immunopositive cells into the neuronal circuitry of the white matter in PVL will ultimately depend upon timing and extent of injury, as well as the availability of neurotropic factors necessary for cellular differentiation and the formation of functional circuits.

## 4. The Bridge between Human and Animal Research in the Encephalopathy of Prematurity

### 4.1. Strengths of Human Neuropathologic Studies in Translational Research in EP

Insights from such studies are crucial to translational research because they shape the relevant hypotheses for animal models by defining the vulnerable cell populations and brain regions, pathogenic molecules, and cellular features of the inflammatory and reparative responses. Human studies have taught us that: (1) pre-OLs, neurons, and axons *in combination* are the key cellular substrates at risk in EP; (2) the cerebral white matter, cerebral cortex, thalamus, basal ganglia, cerebellum, and brainstem are the key brain regions involved; (3) cerebral white matter damage involves micro- and/or macrofoci of necrosis with macrophages in combination with diffuse reactive gliosis and microglial activation and axonal damage; (3) reactive astrocytes and activated microglia are critical components of both gray and white matter injury. Moreover, human neuropathologic studies provide insights into the anatomic substrate of the cognitive, emotional, and behavioral disorders in preterm survivors that are not always forthcoming in animal models, given the profound species differences in executive functions and higher affective processing. Indeed, the spectrum of neuronal and axonal lesions in EP elucidates the basis of the complex *cognitive* deficits in preterm survivors and indicates that these deficits are not based upon white matter damage alone, but rather, likely result from simultaneous damage to diverse nodes in cognitive processing, that is, the corticothalamic-commissural-associative-subplate network [9, 46, 51, 56, 69, 75]. The finding of thalamic damage in the mediodorsal nucleus and reticular nucleus associated with PVL, for example, may help explain the clinical observations of deficits in working memory and state regulation, respectively, in preterm survivors [69], the finding of cerebellar damage helps explain the autistic behaviors [12, 72], and the finding of secondary cerebral cortical changes overlying necrotic white matter lesions, the seizure disorders, and cortical-based cognitive impairments [75]. In addition, the tissue demonstration of neuronal loss and/or gliosis in gray matter sites provides a starting point for establishing the cellular underpinnings of gray matter volume deficits defined by neuroimaging studies [10–12], with the need for future investigations into the potential contributions of associated neuropil (synaptic) loss. The human neuropathologic studies also indicate the intersection of destructive injury and altered developmental trajectories, for example, acquired damage to axons traversing the cerebral white matter to and from the cortex and subsequent trophic neuronal changes in the overlying cortex [46, 75], cerebellar atrophy/underdevelopment [70–72].

### 4.2. Strengths of Studies in Animal Models in Translational Research in EP

Despite their many strengths, human neuropathologic studies have several drawbacks that mandate their performance in unison with animal studies in order to establish the “complete picture” of EP. Indeed, the examination of human tissue sections under the microscope provides only a single snapshot in which the dynamic process is frozen at one single time point and the distinction between primary and secondary features is impossible. Moreover, while the patterns of injury in human tissue sections can suggest a mechanism, for example, coagulative necrosis and ischemia [3], the patterns are not always pathognomonic and therefore cannot specify the mechanism(s) precisely. Thus, animal models are essential for the determination of cellular and molecular mechanisms critical for the development of therapeutic interventions in patient care. Examples include the testing of different drugs in the prevention or amelioration of white matter damage in rodent models [81–84]. The strength of animal models in deciphering mechanisms is well illustrated in studies addressing the relative roles of hypoxia ischemia and infection/inflammation in pre-OL cell death in perinatal white matter damage. In a variety of small and large animal models, hypoxia ischemia has been shown to lead directly to pre-OL damage [81–88]. Yet, several animal models indicate that hypoxia ischemia alone is not always sufficient to cause brain injury, but rather, results in significant injury only when combined with an infectious/inflammatory insult, notably pretreatment with lipopolysaccharide (LPS) [8, 88, 89]. When LPS administration is followed by hypoxia ischemia in a perinatal murine model, for example, pre-OL death occurs acutely and is then followed by decreased mature, MPB-expressing OLs [88]. Chronically administered LPS, however, does not induced hypoxemia in the fetal sheep model but also causes white matter injury, with axonal damage, activated microglia, and OL injury [90], albeit to less severe degrees than when applied acutely [91]. Thus, animal models allow for testing hypotheses about causal factors alone and in combination, the latter more faithfully mimicking the complex clinical course of preterm infants with multiple simultaneous insults.

Nevertheless, animal models also have limitations for mechanistic testing. Cell culture and slice systems are needed to determine the molecular and biochemical effects of injury upon single cell types, as exemplified by the determination of the basis of the vulnerability to glutamate and free radical toxicity of pre-OLs compared to mature (myelinating) OLs [8, 92–95] and the effects of different trophic factors on OL proliferation, differentiation, and myelin sheath synthesis [96].

An additional strength of animal models is that they allow for elucidation of evolution of the histopathologic changes though the sequential examination of brains from a cohort of animals sacrificed at different time points following a common insult [83, 87]. This approach is well demonstrated in the delineation of the sequence of events following uterine artery ligation in a rat model in which cell death, as defined by TUNEL-positivity at P3, was followed by O4 cell loss at P7, microglial activation, and then reactive gliosis in the cerebral white matter, with the persistence of impaired myelination into adulthood [87]. In this way, we learn that apoptosis is involved in pre-OL loss and precedes cell dropout, and that pre-OL damage precedes and therefore potentially incites the inflammatory responses (microglial and then astrocytic activation) in the early stages. Animal models can also lead to novel insights into molecules critical to human lesions but not originally recognized in the lesions *per se*, thereby providing new leads for human investigation. The role of A1 adenosine receptors in the pathogenesis of preterm white matter damage, for example, was not suggested from human neuropathologic studies but rather, from animal models. In a rat model in which hypoxia ischemia leads to pre-OL injury at P3-P12, cerebral hypomyelination was prevented by the administration of caffeine [97]. The postulated mechanism for the caffeine benefit relates to the presence of A1 adenosine receptors on pre-OLs which when activated inhibit pre-OL maturation; caffeine, which blocks A1 adenosine receptors, may in turn remove the maturation block [97]. The relevance of molecules originally discovered in animal lesions to human pathology is determined by their demonstration in human lesions with immunocytochemistry or other applied tissue methods. In this way, animal models “feed back” to the human condition and expand upon its elucidation in new ways. It is important to demonstrate with human tissue methods the expression of adenosine receptors by human pre-OLs to confirm the relevance and ultimate therapeutic potential of adenosine receptor blockage in human PVL. Similarly, the unanticipated observation in the sheep model that the vulnerability of the deep white matter to hypoxia ischemia is related to the increased spatial concentration of a susceptible population of pre-OLs and not to a preferential reduction in cerebral blood flow compared to the intragyral white matter or cortex [98, 99] needs to be pursued in human white matter by studies of the quantitative distribution of pre-OLs in periventricular, deep, and intragyral white matter zones relative to each other to determine the relevance of the animal discovery to human pathogenesis. Nevertheless, these sheep studies corroborate the role of ischemia in the deep white matter to the genesis of pre-OL injury.

### 4.3. Types of Animal Models for Translational Research in EP

Multiple animal models of perinatal brain injury are currently available, generally with the studies focused to date upon white *or* gray matter injury, rarely both in combination. Several comprehensive reviews delineate the pros and cons of the different (small and large) animal models relative to preterm brain injury [96, 100–110]. The strengths of rodent models include a comparable timetable of OL lineage in the cerebral white matter, relative ease and low cost of experimental manipulations, and capability to utilize genetically engineered (knockout) mice. Their disadvantages include the paucity of cerebral white matter and the lack of cortical gyration. Moreover, there are key intrinsic differences in aspects of cerebral development between human and rodent. A relevant example in this context is the origin of GABAergic neurons, for example, nearly entirely from the ganglionic eminence in the rodent but principally from the dorsal pallium in the human [37]. The strengths of large animals, on the other hand, include major structural similarities with the developing human brain, including in gyration and the sequence of OL differentiation, comparable scaling of gestational age relative to brain development, the capability for invasive instrumentation relative to measures of cerebral blood flow and cardiorespiratory parameters, and closer analogy of neurological consequences to those in human preterm infants. Their disadvantages include the need for considerable expertise and resources in large animal husbandry and surgical and supportive procedures.

### 4.4. Caveats in Modeling White Matter Injury in the Preterm Infant

As noted above, the clinical picture of preterm white matter injury is changing such that cystic PVL is now uncommon and has been replaced by a “diffuse” lesion in neuroimaging studies in living infants. Studies in sheep suggest that cystic PVL results from severe ischemic insults, whereas diffuse lesions result from lesser degrees of ischemia [98, 99]; thus, the decline in cystic PVL in the neonatal nursery may reflect in part improvements in the management of the cardiorespiratory disorders of prematurity. Because cystic PVL may indeed represent the severe end of the spectrum, it is nevertheless common in fatal human cases that are presumably the most severely challenged. Still, the presence of cystic PVL at autopsy in the current era cannot be ignored because it indicates that the responsible pathogenic factors in the past era (when cystic PVL was the dominant white matter lesion by neuroimaging) are still operative.

**Figure 1:**
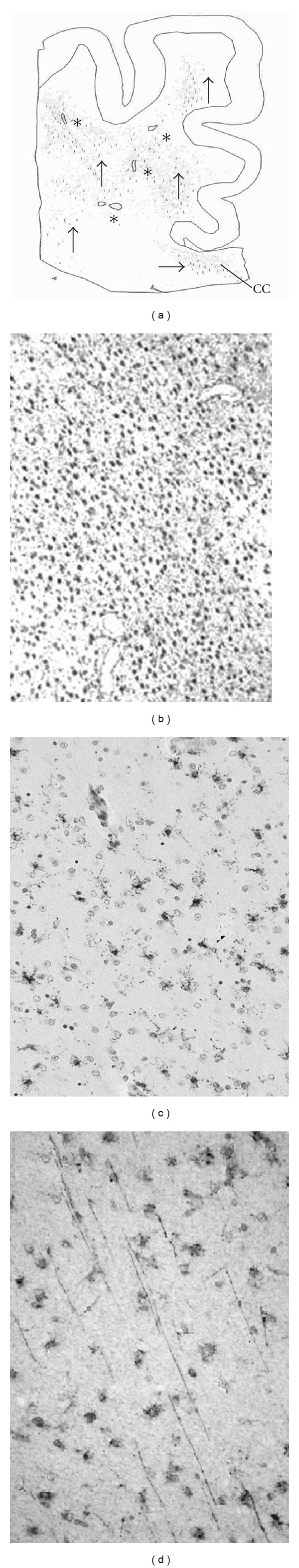
White matter damage in the human preterm brain is characterized by microscopic foci of necrosis and diffuse reactive gliosis, microglial activation, and axonal damage. (a) Camera lucida drawing of the distribution of microcysts (*) and axonal fragments (arrows) in the posterior frontal white matter (level of the body of the corpus callosum [CC]). In the white matter distant from periventricular foci of necrosis is reactive gliosis, as demonstrated by the immunomarker glial fibrillary acidic protein (b), microglial activation, as demonstrated by the immunomarker CD68 (c), and axonal injury, as demonstrated by the immunomarker fraction (d).

What is the neuropathology of the “diffuse” lesion seen by neuroimaging studies in the preterm infant today? Based upon human autopsy studies, this lesion is comprised, in our opinion, of foci of microcystic necrosis in the deep white matter (with these small cysts below the detection capability of modern neuroimaging techniques) in association with diffuse microglial activation, gliosis, and axonal damage (Figure 1). Precise correlations between neuroimaging and autopsy findings in the same infant at the time of death are needed for verification that this microcystic lesion is in fact the diffuse lesion of neuroimaging studies. In a fetal sheep model, however, high-field MRI of chronic perinatal white matter injury indicates correlations between particular patterns of images with microscopic necrosis and reactive gliosis and with pre-OL maturation arrest upon histopathologic examination [111]. In essence, microcystic PVL (with diffuse gliosis and microglial activation) remains a major finding in the preterm brain in active pediatric neuropathology services today. Until proven otherwise, the hallmark of preterm white matter injury remains focal necrosis with macrophages, and its replication in animal models, as well as its relationship to pre-OL injury, should be sought in translational research.

### 4.5. The Selection of an Animal Model in EP

The question arises: should an animal paradigm model the entire neuropathologic spectrum of EP and thereby cell interactions in pathogenesis, or rather, model one feature of the spectrum, for example, pre-OL cell death, in search of a cell-specific mechanism? The answer is obviously that both types of models play valuable and complementary roles. Yet, it is increasingly clear over the last decade from neuroimaging and neuropathologic studies that human preterm injury is a complex spectrum of pre-OL, neuronal, and axonal injury in multiple brain regions, as well as distinct inflammatory responses, reparative events, hemorrhages, and focal infarcts [2, 3, 6], and the interrelationships of these pathologic processes need to be determined to elucidate the shared mechanisms and sequential cascade of the tissue reactions. It is critical, for example, to examine pre-OL and axonal damage in conjunction with each other to determine the molecular influences of injured pre-OLs and axons upon each other in the initiation and progression of myelin sheath wrapping. While human studies indicate both focal and diffuse axonal injury in PVL [7, 62, 73, 74], such injury is often not addressed in animal models in the same brains that undergo intensive investigations of pre-OLs. In a recent neonatal rat model of hypoxia ischemia in which axonal integrity was assessed with the antineurofilament antibody SMI-312, axonal degeneration or reductions in axonal density were not observed, whereas pre-OLs were present that failed to initiate myelination [86]. The apparent resistance of the axons to hypoxic ischemic injury in this model was likened to that of murine axons at P3 in an oxygen glucose deprivation model [112]. In a large animal (sheep) model, however, axonal degeneration was apparent with prolonged ischemia [98], providing insight into the potential basis of the widespread axonal damage in human preterm infants [73].

Still, the major focus in animal models related to preterm brain damage to date has been upon white matter injury and the cellular mechanisms of pre-OL injury almost in isolation for a variety of historical reasons, as recently reviewed [4]. This focus illustrates in large part the cell-specific approach to animal models, as they address the hypothesis that hypoxia-ischemia causes pre-OL cell death and hypomyelination. The major criteria for an animal model of white matter injury are thus a cellular sequence of OL lineage similar to that of human preterm white matter and an analogous developmental window when pre-OLs dominate. Various hypoxic-ischemic models have demonstrated the death and dropout of pre-OLs with O1 and O4 immunomarkers, the regeneration and arrested maturation of pre-OLs with proliferative and early OL markers, and hypomyelination by loss of MBP staining [81, 85, 86, 88, 97–99]. Thus, these models have successfully delineated the “natural history” of pre-OL damage upon exposure to hypoxia ischemia and have established unequivocally the role of this insult in white matter injury. Moreover, they have provided an important explanation for the observation in human PVL that the density of OLs labeled with the immunomarker OLIG2 is not decreased, that is, this marker labels all stages of OL lineage and therefore this preservation of OL density may reflect proliferation of pre-OLs with arrested maturation with dominance of pre-OLs over mature OLs [66]. Animal models have also solidified the role of glutamate and free radicals in the tissue injury. In a preterm fetal sheep model of bilateral carotid occlusion, for example, extracellular excitatory aminoacids and malondialdehyde (but not 8-isoprostane) were significantly increased in the periventricular white matter with a peak at 2-3 days following occlusion [113].

Yet, the pathology of white matter damage in the human infant is far more complex than pre-OL damage only. Indeed, the hallmark of PVL is focal necrosis with macrophages in association with diffuse reactive gliosis and microglial activation [3, 7], and it is possible that different mechanisms are operative in producing pre-OL damage *and* focal necrosis as opposed to diffuse pre-OL damage *without* focal necrosis, as seen in certain animal models (e.g., [81]). Given that microglial activation is a hallmark of PVL, the role of microglia relative to pre-OL injury is of critical interest. The demonstration that the drug minocycline suppresses microglial activation and substantially attenuates pre-OL injury in perinatal rodent models of hypoxia ischemia is important [83, 84]. It underscores the role of microglia in the pathogenesis of PVL, as suggested by focal macrophagocytic infiltration and diffuse microglial activation in surrounding nonnecrotic white matter [64], and provides a potential effective means of intervention [83, 84]. Microglia mediate pre-OL cell death, at least in part, via pathways of oxidative and nitrative stress [84].

Indeed, it is likely that macro- and microcysts in the white matter result when the ischemic insult is severe enough to cause concomitant gray matter injury, as suggested by the finding that gray matter neuronal loss and/or gliosis in the human preterm infant occurs only in association with necrotic foci and not with gliosis alone [9] and that prolonged ischemia in the sheep model results in white *and* gray matter pathology [98]. In the past, animal models have been sought that demonstrate white matter injury exclusively, and those with both gray and white matter injury were considered undesirable. It is likely, however, that in the effort to “create” only white matter injury (without associated gray matter injury), that is, the historically perceived dominant pathology of the preterm infant [4], animal models were based upon lesser degrees of insult that “stopped short” of (white or gray matter) necrosis, and the insult was not severe enough to recapitulate the entire spectrum of the human pathology. The challenge in animal modeling now is to discover the timing, degree, and type of insult that recapitulate the full human spectrum if further advances, in our opinion, are to be made.

The approach to modeling the whole spectrum of EP is indeed complicated, and may not be possible, given the complexity of the histopathology, the nonspecificity of certain lesions, for example, neuronal loss and gliosis, the likelihood that multiple insults are involved for example, ischemia, infection, hemorrhage, hyperbilirubinemia, and hypoglycemia, and the variable timing (e.g., intermittent, recurrent) and intensity of the insults. The analysis of the thalamus in association with PVL indicates heterogeneous lesions implicating different mechanisms, for example, diffuse gliosis and neuronal loss consistent with generalized hypoxia ischemia, microinfarcts consistent with small arterial vessel thrombi, and large infarcts consistent with large (posterior arterial) occlusions [69]. The underdevelopment of the cerebellum in the preterm infant may not be directly related to hypoxia ischemia but rather to a secondary consequence of intraventricular hemorrhage, heme deposition in the leptomeninges, and heme toxicity to the external granular layer with secondary cell loss and impaired migration to the internal granular layer [72].

One approach to analyzing the whole spectrum of EP is to focus upon animal models that mimic the circumstances of prematurity in the modern neonatal intensive care nursery without a specific single severe insult. In this regard, the baboon model of preterm delivery and subsequent care with mechanical ventilation, blood gas and electrolyte monitoring, and administration of pressors indicates a spectrum of white matter injury, including focal necrosis, gray matter (hippocampus) injury, focal and leptomeningeal hemorrhages, and ventriculomegaly that in multiple respects mirrors human EP [108]. This model allows for the determination of the natural history of injury to pre-OLs under the nearly identical circumstances that most closely reflect that of the human preterm infant. While this baboon model does not provide the unequivocal establishment of the specific mechanism of pre-OL cell death, it allows for the determination of the sequence of pre-OL cell injury in the setting of the multiple insults of human prematurity and intensive management.

 Animal models of EP need to focus upon *combined* gray and white matter injury to facilitate the discovery of shared cellular and molecular pathways that lead to pre-OL, axonal, and neuronal damage that are all seen in the single “snapshot” at tone time under the microscope, having occurred simultaneously or at different times in the newborn's clinical course. Developing neurons, axons, and OLs, for example, are known to share molecular pathways leading to apoptosis and thus the development of a drug that targets these pathways could potentially prevent neuronal and pre-OL cell death at the same time. Shared pathways may relate also to glutamate receptors and free radical defenses, as these factors involve both pre-OL and neuronal toxicity [8]. Both cell types, for example, express glutamate receptor subtypes in the human preterm brain that mediate excitotoxicity [27–29], and animal models that test glutamate receptor antagonists need to assess protection of both white and gray matter populations. Alternatively, drug testing in animal models may need to provide an agent that targets pre-OL injury and one that targets neuronal injury at the same time. Indeed, combined models that delineate mechanistic commonalities between pre-OLs, neurons, and axons may yield synergistic therapeutic agents that prove to be the most effective in preventing the *global* consequences of EP. The sheep model, for example, demonstrates white matter injury in conjunction with basal ganglia and cortical injury [98], and the elucidation of the mechanisms underlying these combined lesions could indeed be sought in this key model.

### 4.6. Synergy between Human Neuropathologic and Animal Models Studies in Translational Research in EP

Translational research is advanced by the analysis of human and animal studies in parallel, with each approach informing the other. This vital synergy is well illustrated by the recent study of the role of ceramide, a bioactive sphingolipid pivotal to sphingolipid metabolism pathways, in PVL in which parallel human and animal analyses were presented in a single comprehensive publication [114]. Ceramide, which regulates cell death in response to diverse stimuli, was found to accumulate in reactive astrocytes in the diffuse component of human PVL by immunocytochemical methods, thereby establishing it as a factor in the human pathology [114]. Next, ceramide was reported in cell culture to interact with the cytokine tumor necrosis factor, resulting in apoptotic death of OLs in an astrocyte-dependent manner. Finally, altered sphingolipid metabolism was restored during spontaneous remyelination following toxic-induced demyelination in a whole animal model. Taken together, these studies suggest that the modulation of sphingolipid signaling pathways in reactive astrocytes is a potentially important and novel means to prevent PVL in humans [114]. The demonstration of ceramide accumulation in reactive astrocytes in PVL solidified the relevance of the experimental findings to the human condition. A second example of the synergistic value of human, whole animal, and cell culture models concerns the presence of GluR2 AMPA-deficient receptors and NMDA receptors on pre-OLs and the protection afforded by respectively topiramate and memantine against excitotoxicity [81, 82]. In addition, the discovery of diffuse microglial activation in the white matter surrounding necrotic foci led to a body of experimental data demonstrating the role of microglia in innate immunity in microglial activation, toll-like receptor biology, necrotic reactions, cytokine production, and free radical generation [8, 94, 115, 116], with the potential therapeutic relevance of the amelioration by minocycline of white matter damage in animal models [81, 82]. The synthesis of the human and animal data leads in turn to the provocative insight that microglia are the critical “convergence point” in the potentiation of hypoxic ischemic and infectious/inflammatory insults in PVL, as recently reviewed in depth [8].

## 5. Conclusions

The pathology of EP is complex and heterogeneous and mandates multiple types of large and small animal models to address all of its many facets in global and cell-specific paradigms. It could be argued that the “best” animal in which to model EP is the animal in which EP occurs in the natural state, as in the report of PVL in neonatal monkeys born prematurely [117]: here all of the “right”, human-like, factors must be in place, operative, and spontaneous. While no one experimental model captures all of the complexity of the human disorder, important advances in our understanding of preterm brain injury have resulted from different experimental approaches that focus on different questions, resulting in an increasingly complete picture. In tandem with animal models are the human neuropathologic studies with state-of-the-art methods, including gene expression profiling [49], proteomics [118], western blotting [29, 30, 41, 44, 50, 52], stereology [119], array tomography [120], immunoprecipitation and protein identification [42], tissue receptor autoradiography [27, 28, 50, 58, 59], single- and double-label immunocytochemistry [22, 23, 29, 38, 64], biochemical assays [65], histochemistry [30, 39], electron microscopy [39, 40], and confocal microscopy [22], as well as the ever-valuable Golgi technique [16, 40, 48, 75]. Yet, at this time of unprecedented tools for human brain analysis, the autopsy rates are unacceptably low. We urgently need to develop a culture among those caring for premature infants that place supreme value upon the role of the autopsy in research so that families are readily and routinely approached for consent. Central tissue banks have also been advocated to facilitate preterm brain research given the difficulties for any one single investigator to accrue sufficient sample sizes [96]. In addition, the scientific community at large needs to place a premium on the unique role of human neuropathologic studies in translational research, with an appreciation of the applicability of highly sophisticated and quantitative tools for tissue analysis for which the effects of postmortem can be corrected. Rather than downplaying human autopsy-based research as not “mechanism driven” or “hypothesis testing”, the scientific community should value such investigation for its many strengths, specifically the role in defining the major cell types, brain regions, and molecules in the human condition and generating relevant hypotheses for mechanistic testing in experimental systems. In short, human and animal studies in parallel are essential to inform and build upon each other; one without the other just won't work.

## Figures and Tables

**Table 1:** Key developmental events in the cerebral white matter, cortex, and subplate region in the last half of human gestation for considering in the design of animal models of the encephalopathy of prematurity.

(1) Cerebral white matter	
(a) Development of vasculature and autoregulation	
(b) Dominance of pre-OLs	
(c) Overexpression of pre-OLs of calcium-permeable, GluR2-deficient AMPA receptors	
(d) Expression of pre-OLs of NDMA receptors	
(e) Transient expression of glutamate transporter EAAT2	
(f) Transient abundance of microglia	
(g) Oligodendrocyte expression of cytokine (interferon-*γ*) receptors	
(h) Radial glial fiber transformation and disappearance	
(i) Late formation of fibrous astrocytes	
(j) Lag in the expression of superoxide dismutases	
(k) Active axonal elongation	
(2) Cerebral cortex	
(a) Gyration	
(b) Lamination	
(c) Neuronal differentiation	
(d) Late migration of GABAergic neurons	
(e) Late formation of protoplasmic astrocytes following neuronal migration	
(3) Subplate region	
(a) Ingrowth of axons and “waiting period”	
(b) Involution	

**Table 2:** Major histopathology features of the encephalopathy of prematurity in the human brain.

(1) White matter	
(a) Periventricular leukomalacia of the telencephalic white matter	
(i) Periventricular focal necrosis in different stages (acute, organizing, and macro- and/or microcysts)	
(ii) Gliosis and microglial activation in the surrounding white matter	
(iii) Early loss of pre-OLs	
(iv) Expression of markers of oxidative and nitrative stress by pre-OLs	
(v) Possible maturation arrest of OLs	
(vi) Impaired myelin formation	
(vii) Upregulation of cytokines in macrophages, activated microglia, and reactive astrocytes	
(b) Widespread axonal damage within and distant from the necrotic foci	
(c) Deficit of neurons within necrotic foci, surrounding white matter distant from the necrotic foci, and subplate region	
(d) Postmitotic migrating neurons as possible reparative event	
(e) Gliosis of the cerebellar white matter	
(2) Gray matter	
(a) Neuronal loss and/or gliosis of the cerebral cortex, thalamus, globus pallidus, hippocampus, cerebellum, and brainstem in different combinations and to different degrees, with preferential involvement of thalamus and basal ganglia	
(3) Hemorrhages	
(a) Subpial	
(b) Subarachnoid	
(c) Germinal matrix (with suppression of cell proliferation)	
(d) Cerebellum	
(4) Infarcts	
(a) Microinfarcts of the thalamus	
(b) Focal infarcts of the cerebral cortex	

## Abbreviations

AMPA: Alpha-amino-3-hydroxy-5-methyl-4-isoxazole-propionic acid
DCX: Doublecortin
EAAT: Excitatory aminoacid transporter
EAAT1: Excitatory aminoacid transporter 1
EAAT2: Excitatory aminoacid transporter 2
EP: Encephalopathy of prematurity
GABA: *γ*-aminobutyric acid
GAP-43: Growth-associated protein-43
GFAP: Glial fibrillary acidotic protein
GluR2: Glutamate receptor subunit 2
KCC1: Potassium chloride cotransporter 1
LPS: Lipopolysaccharide
MPB: Myelin basic protein
NG2: Proteoglycan 2
NKCC1: Sodium potassium chloride cotransporter 1
NMDA: N-methyl-D-aspartate
OL: Oligodendrocyte
Pre-OL: Premyelinating oligodendrocyte
PVL: Periventricular leukomalacia
RGF: Radial glial fiber.

## References

[1] Volpe JJ (2005). Encephalopathy of prematurity includes neuronal abnormalities. *Pediatrics*.

[2] Volpe JJ (2009). Brain injury in premature infants: a complex amalgam of destructive and developmental disturbances. *The Lancet Neurology*.

[3] Kinney HC, Volpe JJ, Haddad GG, Yu SP (2009). Perinatal panencephalopathy in premature infants: is it due to hypoxia-ischemia?. *Brain Hypoxia and Ischemia with Special Emphasis on Development*.

[4] Kinney HC (2009). The encephalopathy of prematurity: one pediatric neuropathologist’s perspective. *Seminars in Pediatric Neurology*.

[5] Del Bigio MR (2011). Cell proliferation in human ganglionic eminence and suppression after prematurity-associated haemorrhage. *Brain*.

[6] Armstrong DL, Sauls CD, Goddard-Finegold J (1987). Neuropathologic findings in short-term survivors of intraventricular hemorrhage. *American Journal of Diseases of Children*.

[7] Folkerth RD, Kinney HC, Louis D (2009). Perinatal Neuropathology. *Greenfield’s Neuropathology*.

[8] Volpe JJ, Kinney HC, Jensen FE, Rosenberg PA (2011). The developing oligodendrocyte: key cellular target in brain injury in the premature infant. *International Journal of Developmental Neuroscience*.

[9] Pierson CR, Folkerth RD, Billiards SS (2007). Gray matter injury associated with periventricular leukomalacia in the premature infant. *Acta Neuropathologica*.

[10] Peterson BS, Vohr B, Staib LH (2000). Regional brain volume abnormalities and long-term cognitive outcome in preterm infants. *JAMA*.

[11] Isaacs EB, Lucas A, Chong WK (2000). Hippocampal volume and everyday memory in children of very low birth weight. *Pediatric Research*.

[12] Limperopoulos C, Bassan H, Sullivan NR (2008). Positive screening for autism in ex-preterm infants: prevalence and risk factors. *Pediatrics*.

[13] Lee JD, Park HJ, Park ES (2011). Motor pathway in injury in patients with periventricular leukomalacia and spastic diplegia. *Brain*.

[14] Fazzi E, Bova S, Giovenzana A, Signorini S, Uggetti C, Bianchi P (2009). Cognitive visual dysfunctions in preterm children with periventricular leukomalacia. *Developmental Medicine and Child Neurology*.

[15] Sidman RL, Rakic P, Adams RD, Haymaker W (1973). Development of the human central nervous system. *Cytology and Cellular Neuropathology*.

[16] Marin-Padilla M (2010). *The Human Brain: Prenatal Development and Structure*.

[17] Volpe JJ (2008). *Neurology of the Newborn*.

[18] Takashima S, Tanaka K (1978). Development of cerebrovascular architecture and its relationship to periventricular leukomalacia. *Archives of Neurology*.

[19] Tsuji M, Saul JP, Du Plessis A (2000). Cerebral intravascular oxygenation correlates with mean arterial pressure in critically ill premature infants. *Pediatrics*.

[20] Soul JS, Hammer PE, Tsuji M (2007). Fluctuating pressure-passivity is common in the cerebral circulation of sick premature infants. *Pediatric Research*.

[21] Back SA, Luo NL, Borenstein NS, Levine JM, Volpe JJ, Kinney HC (2001). Late oligodendrocyte progenitors coincide with the developmental window of vulnerability for human perinatal white matter injury. *Journal of Neuroscience*.

[22] Back SA, Luo NL, Borenstein NS, Volpe JJ, Kinney HC (2002). Arrested oligodendrocyte lineage progression during human cerebral white matter development: dissociation between the timing of progenitor differentiation and myelinogenesis. *Journal of Neuropathology and Experimental Neurology*.

[23] Jakovcevski I, Filipovic R, Mo Z, Rakic S, Secevic N (2009). Oligodendrocyte development and the onset of myelination in the human fetal brain. *Frontiers in Neuroanatomy*.

[24] Jakovcevski I, Zecevic N (2005). Olig transcription factors are expressed in oligodendrocyte and neuronal cells in human fetal CNS. *Journal of Neuroscience*.

[25] Kinney HC, Brody BA, Kloman AS, Gilles FH (1988). Sequence of central nervous system myelination in human infancy. II. Patterns of myelination in autopsied infants. *Journal of Neuropathology and Experimental Neurology*.

[26] Haynes RL, Kinney HC (2011). *Central Axonal Development and Pathology in Early Life. Handbook of Neurochemistry and Molecular Neurobiology*.

[27] Lee H, Choi BH (1992). Density and distribution of excitatory amino acid receptors in the developing human fetal brain: a quantitative autoradiographic study. *Experimental Neurology*.

[28] Greenamyre T, Penney JB, Young AB (1987). Evidence for transient perinatal glutamatergic innervation of globus pallidus. *Journal of Neuroscience*.

[29] Talos DM, Follett PL, Folkerth RD (2006). Developmental regulation of *α*-amino-3-hydroxy-5-methyl-4-isoxazole- propionic acid receptor subunit expression in forebrain and relationship to regional susceptibility to hypoxic/ischemic injury. II. Human cerebral white matter and cortex. *Journal of Comparative Neurology*.

[30] Desilva TM, Kinney HC, Borenstein NS (2007). The glutamate transporter EAAT2 is transiently expressed in developing human cerebral white matter. *Journal of Comparative Neurology*.

[31] Monier A, Adle-Biassette H, Delezoide A-L, Evrard P, Gressens P, Verney C (2007). Entry and distribution of microglial cells in human embryonic and fetal cerebral cortex. *Journal of Neuropathology and Experimental Neurology*.

[32] Monier A, Evrard P, Gressens P, Verney C (2006). Distribution and differentiation of microglia in the human encephalon during the first two trimesters of gestation. *Journal of Comparative Neurology*.

[33] Billiards SS, Haynes RL, Folkerth RD (2006). Development of microglia in the cerebral white matter of the human fetus and infant. *Journal of Comparative Neurology*.

[34] Folkerth RD, Keefe RJ, Haynes RL, Trachtenberg FL, Volpe JJ, Kinney HC (2004). Interferon-*γ* expression in periventricular leukomalacia in the human brain. *Brain Pathology*.

[35] Gray GE, Sanes JR (1992). Lineage of radial glia in the chicken optic tectum. *Development*.

[36] Rubenstein JLR (2011). Annual research review: development of the cerebral cortex: implications for neurodevelopmental disorders. *Journal of Child Psychology and Psychiatry and Allied Disciplines*.

[37] Letinic K, Rakic P (2001). Telencephalic origin of human thalamic GABAergic neurons. *Nature Neuroscience*.

[38] deAzevedo LC, Fallet C, Moura-Neto V, Daumas-Duport C, Hedin-Pereira C, Lent R (2003). Cortical radial glial cells in human fetuses: depth-correlated transformation into astrocytes. *Journal of Neurobiology*.

[39] Kadhim HJ, Gadisseux J-F, Evrard P (1988). Topographical and cytological evolution of the glial phase during prenatal development of the human brain: histochemical and electron microscopic study. *Journal of Neuropathology and Experimental Neurology*.

[40] Choi BH, Lapham LW (1978). Radial glia in the human fetal cerebrum: a combined Golgi, immunofluorescent and electron microscopic study. *Brain Research*.

[41] Folkerth RD, Haynes RL, Borenstein NS (2004). Developmental lag in superoxide dismutases relative to other antioxidant enzymes in premyelinated human telencephalic white matter. *Journal of Neuropathology and Experimental Neurology*.

[42] Haynes RL, Folkerth RD, Szweda LI, Volpe JJ, Kinney HC (2006). Physiological lipid peroxidation during the period of active myelination. *Journal of Neuropathology & Experimental Neurology*.

[43] Kostoví I, Judaš M (2002). Correlation between the sequential ingrowth of afferents and transient patterns of cortical lamination in preterm infants. *Anatomical Record*.

[44] Haynes RL, Borenstein NS, Desilva TH (2005). Axonal development in the cerebral white matter of the human fetus and infant. *Journal of Comparative Neurology*.

[45] Judaš M, Radoš M, Jovanov-Miloševic N, Hrabac P, Štern-Padovan R, Kostovic I (2005). Structural, immunocytochemical, and MR imaging properties of periventricular crossroads of growing cortical pathways in preterm infants. *American Journal of Neuroradiology*.

[46] Andiman SE, Haynes RL, Trachtenberg FL (2010). The cerebral cortex overlying periventricular leukomalacia: analysis of pyramidal neurons. *Brain Pathology*.

[47] Kostovic I (1990). Structural and histochemical reorganization of the human prefrontal cortex during perinatal and postnatal life. *Progress in Brain Research*.

[48] Becker LE, Armstrong DL, Chan F, Wood MM (1984). Dendritic development in human occipital cortical neurons. *Brain Research*.

[49] Johnson MB, Kawasawa YI, Mason CE (2009). Functional and evolutionary insights into human brain development through global transcriptome analysis. *Neuron*.

[50] Xu G, Broadbelt KG, Haynes RL (2011). Late development of the gabaergic system in the human cerebral cortex and white matter. *Journal of Neuropathology and Experimental Neurology*.

[51] Robinson S, Li Q, DeChant A, Cohen ML (2006). Neonatal loss of *γ*-aminobutyric acid pathway expression after human perinatal brain injury. *Journal of Neurosurgery*.

[52] Dzhala VI, Talos DM, Sdrulla DA (2005). NKCC1 transporter facilitates seizures in the developing brain. *Nature Medicine*.

[53] DeSilva TM, Borenstein NS, Volpe JJ, Kinney HC, Rosenberg PA Differential expression of the excitatory amino acid transporters EAAT1-3 in the developing human cerebral cortex.

[54] Kostovic I, Rakic P (1990). Developmental history of the transient subplate zone in the visual and somatosensory cortex of the macaque monkey and human brain. *Journal of Comparative Neurology*.

[55] Kanold PO, Luhmann HJ (2010). The subplate and early cortical circuits. *Annual Review of Neuroscience*.

[56] Kinney HC, Haynes RL, Xu G (2012). Neuron deficit in the white matter and subplate in periventricular leukomalacia. *Annals of Neurology*.

[57] Suarez-Sola M, Gonzalez-Delgado J, Pueyo-Morlans M (2009). Neurons in the white matter of the adult human neocortex. *Frontiers in Neuroanatomy*.

[58] Panigrahy A, Rosenberg PA, Assmann S, Foley EC, Kinney HC (2000). Differential expression of glutamate receptor subtypes in human brainstem sites involved in perinatal hypoxia-ischemia. *Journal of Comparative Neurology*.

[59] Panigrahy A (1995). Developmental changes in [3H]kainate binding in human brainstem sites vulnerable to perinatal hypoxia-ischemia. *Neuroscience*.

[60] Takikawa M, Kato S, Esumi H (2001). Temporospatial relationship between the expressions of superoxide dismutase and nitric oxide synthase in the developing human brain: immunohistochemical and immunoblotting analyses. *Acta Neuropathologica*.

[61] Banker BQ, Larroche JC (1962). Periventricular leukomalacia of infancy. A form of neonatal anoxic encephalopathy. *Archives of Neurology*.

[62] Haynes RL, Billiards SS, Borenstein NS, Volpe JJ, Kinney HC (2008). Diffuse axonal injury in periventricular leukomalacia as determined by apoptotic marker fractin. *Pediatric Research*.

[63] Leviton A, Gilles FH (1984). Acquired perinatal leukoencephalopathy. *Annals of Neurology*.

[64] Haynes RL, Folkerth RD, Keefe RJ (2003). Nitrosative and oxidative injury to premyelinating oligodendrocytes in periventricular leukomalacia. *Journal of Neuropathology and Experimental Neurology*.

[65] Back SA, Luo NL, Mallinson RA (2005). Selective vulnerability of preterm white matter to oxidative damage defined by F2-isoprostanes. *Annals of Neurology*.

[66] Billiards SS, Haynes RL, Folkerth RD (2008). Myelin abnormalities without oligodendrocyte loss in periventricular leukomalacia. *Brain Pathology*.

[67] Iida K, Takashima S, Ueda K (1995). Immunohistochemical study of myelination and oligodendrocyte in infants with periventricular leukomalacia. *Pediatric Neurology*.

[68] Golden JA, Gilles FH, Rudelli R, Leviton A (1997). Frequency of neuropathological abnormalities in very low birth weight infants. *Journal of Neuropathology and Experimental Neurology*.

[69] Ligam P, Haynes RL, Folkerth RD (2009). Thalamic damage in periventricular leukomalacia: novel pathologic observations relevant to cognitive deficits in survivors of prematurity. *Pediatric Research*.

[70] Limperopoulos C, Soul JS, Haidar H (2005). Impaired trophic interactions between the cerebellum and the cerebrum among preterm infants. *Pediatrics*.

[71] Limperopoulos C, Soul JS, Gauvreau K (2005). Late gestation cerebellar growth is rapid and impeded by premature birth. *Pediatrics*.

[72] Volpe JJ (2009). Cerebellum of the premature infant: rapidly developing, vulnerable, clinically important. *Journal of Child Neurology*.

[73] Haynes RL, Folkerth RD, Trachtenberg FL, Volpe JJ, Kinney HC (2009). Nitrosative stress and inducible nitric oxide synthase expression in periventricular leukomalacia. *Acta Neuropathologica*.

[74] Deguchi K, Oguchi K, Matsuura N, Armstrong DD, Takashima S (1999). Periventricular leukomalacia: relation to gestational age and axonal injury. *Pediatric Neurology*.

[75] Marín-Padilla M (1997). Developmental neuropathology and impact of perinatal brain damage. II: white matter lesions of the neocortex. *Journal of Neuropathology and Experimental Neurology*.

[76] Kadhim H, Tabarki B, Verellen G, De Prez C, Rona AM, Sébire G (2001). Inflammatory cytokines in the pathogenesis of periventricular leukomalacia. *Neurology*.

[77] Popko B, Baerwald KD (1999). Oligodendroglial response to the immune cytokine interferon gamma. *Neurochemical Research*.

[78] Desilva TM, Billiards SS, Borenstein NS (2008). Glutamate transporter EAAT2 expression is up-regulated in reactive astrocytes in human periventricular leukomalacia. *Journal of Comparative Neurology*.

[79] Okoshi Y, Mizuguchi M, Itoh M, Oka A, Takashima S (2007). Altered nestin expression in the cerebrum with periventricular leukomalacia. *Pediatric Neurology*.

[80] Haynes RL, Xu G, Folkerth RD, Trachtenberg FL, Volpe JJ, Kinney HC (2011). Potential neuronal repair in cerebral white matter injury in the human neonate. *Pediatric Research*.

[81] Follett PL, Deng W, Dai W (2004). Glutamate receptor-mediated oligodendrocyte toxicity in periventricular leukomalacia: a protective role for topiramate. *Journal of Neuroscience*.

[82] Manning SM, Talos DM, Zhou C (2008). NMDA receptor blockade with memantine attenuates white matter injury in a rat model of periventricular leukomalacia. *Journal of Neuroscience*.

[83] Lechpammer M, Manning SM, Samonte F (2008). Minocycline treatment following hypoxic/ischaemic injury attenuates white matter injury in a rodent model of periventricular leucomalacia. *Neuropathology and Applied Neurobiology*.

[84] Cai Z, Lin S, Fan LW, Pang Y, Rhodes PG (2006). Minocycline alleviates hypoxic-ischemic injury to developing oligodendrocytes in the neonatal rat brain. *Neuroscience*.

[85] Back SA, Han BH, Luo NL (2002). Selective vulnerability of late oligodendrocyte progenitors to hypoxia-ischemia. *Journal of Neuroscience*.

[86] Segovia KN, McClure M, Moravec M (2008). Arrested oligodendrocyte lineage maturation in chronic perinatal white matter injury. *Annals of Neurology*.

[87] Olivier P, Baud O, Evrard P, Gressens P, Verney C (2005). Prenatal ischemia and white matter damage in rats. *Journal of Neuropathology and Experimental Neurology*.

[88] Wang LW, Chang YC, Lin CY, Hong JS, Huang CC (2010). Low-dose lipopolysaccharide selectively sensitizes hypoxic ischemia-induced white matter injury in the immature brain. *Pediatric Research*.

[89] Eklind S, Mallard C, Leverin AL (2001). Bacterial endotoxin sensitizes the immature brain to hypoxic-ischaemic injury. *European Journal of Neuroscience*.

[90] Duncan JR, Cock ML, Suzuki K, Scheerlinck JPY, Harding R, Rees SM (2006). Chronic endotoxin exposure causes brain injury in the ovine fetus in the absence of hypoxemia. *Journal of the Society for Gynecologic Investigation*.

[91] Duncan JR, Cock ML, Scheerlinck JPY (2002). White matter injury after repeated endotoxin exposure in the preterm ovine fetus. *Pediatric Research*.

[92] Deng W, Pleasure J, Pleasure D (2008). Progress in periventricular leukomalacia. *Archives of Neurology*.

[93] Dean JM, Riddle A, Maire J (2011). An organotypic slice culture model of chronic white matter injury with maturation arrest of oligodendrocyte progenitors. *Molecular Neurodegeneration*.

[94] Li J, Ramenaden ER, Peng J, Koito H, Volpe JJ, Rosenberg PA (2008). Tumor necrosis factor *α* mediates lipopolysaccharide-induced microglial toxicity to developing oligodendrocytes when astrocytes are present. *Journal of Neuroscience*.

[95] Deng W, Yue Q, Rosenberg PA, Volpe JJ, Jensen FE (2006). Oligodendrocyte excitotoxicity determined by local glutamate accumulation and mitochondrial function. *Journal of Neurochemistry*.

[96] Silbereis JC, Huang EJ, Back SA, Rowitch DH (2010). Towards improved animal models of neonatal white matter injury associated with cerebral palsy. *DMM Disease Models and Mechanisms*.

[97] Back SA, Craig A, Luo NL (2006). Protective effects of caffeine on chronic hypoxia-induced perinatal white matter injury. *Annals of Neurology*.

[98] McClure MM, Riddle A, Manese M (2008). Cerebral blood flow heterogeneity in preterm sheep: lack of physiologic support for vascular boundary zones in fetal cerebral white matter. *Journal of Cerebral Blood Flow and Metabolism*.

[99] Riddle A, Ning LL, Manese M (2006). Spatial heterogeneity in oligodendrocyte lineage maturation and not cerebral blood flow predicts fetal ovine periventricular white matter injury. *Journal of Neuroscience*.

[100] Rees S, Harding R, Walker D (2011). The biological basis of injury and neuroprotection in the fetal and neonatal brain. *International Journal of Developmental Neuroscience*.

[101] Shen Y, Plane JM, Deng W (2010). Mouse models of periventricular leukomalacia. *Journal of Visualized Experiments*.

[102] Gressens P, Le Verche V, Fraser M (2011). Pitfalls in the quest of neuroprotectants for the perinatal brain. *Developmental Neuroscience*.

[103] Hagberg H, Bona E, Gilland E, Puka-Sundvall M (1997). Hypoxia-ischaemia model in the 7-day-old rat: possibilities and shortcomings. *Acta Paediatrica, International Journal of Paediatrics, Supplement*.

[104] Scafidi J, Fagel DM, Ment LR, Vaccarino FM (2009). Modeling premature brain injury and recovery. *International Journal of Developmental Neuroscience*.

[105] Vannucci RC, Vannucci SJ (2005). Perinatal hypoxic-ischemic brain damage: evolution of an animal model. *Developmental Neuroscience*.

[106] Hagberg H, Ichord R, Palmer C, Yager JY, Vannucci SJ (2002). Animal models of developmental brain injury: relevance to human disease—a summary of the panel discussion from the Third Hershey Conference on developmental cerebral blood flow and metabolism. *Developmental Neuroscience*.

[107] Hagberg H, Peebles D, Mallard C (2002). Models of white matter injury: comparison of infectious, hypoxic-Ischemic, and excitotoxic insults. *Mental Retardation and Developmental Disabilities Research Reviews*.

[108] Inder T, Neil J, Kroenke C, Dieni S, Yoder B, Rees S (2005). Investigation of cerebral development and injury in the prematurely born primate by magnetic resonance imaging and histopathology. *Developmental Neuroscience*.

[109] Back SA, Riddle A, Hohimer AR (2006). Role of instrumented fetal sheep preparations in defining the pathogenesis of human periventricular white-matter injury. *Journal of Child Neurology*.

[110] Johnston MV, Ferriero DM, Vannucci SJ, Hagberg H (2005). Models of cerebral palsy: which ones are best?. *Journal of Child Neurology*.

[111] Riddle A, Dean J, Buser JR (2011). Histopathological correlates of magnetic resonance imaging-defined chronic perinatal white matter injury. *Annals of Neurology*.

[112] McCarran WJ, Goldberg MP (2007). White matter axon vulnerability to AMPA/kainate receptor-mediated ischemic injury is developmentally regulated. *Journal of Neuroscience*.

[113] Fraser M, Bennet L, Van Zijl PL (2008). Extracellular amino acids and lipid peroxidation products in periventricular white matter during and after cerebral ischemia in preterm fetal sheep. *Journal of Neurochemistry*.

[114] Kim S, Steelman AJ, Zhang Y, Kinney HC, Li J (2012). Aberrant upregulation of astroglial ceramide potentiates oligodendrocyte injury. *Brain Pathology*.

[115] Carlsson Y, Schwendimann L, Vontell R (2011). Genetic inhibition of caspase-2 reduces hypoxic-ischemic and excitotoxic neonatal brain injury. *Annals of Neurology*.

[116] Lehnard S, Lachance C, Patrizi S (2002). The toll-like receptor TLR4 is necessary for lipopolysaccharide-induced oligodendrocyte injury in the CNS. *Journal of Neuroscience*.

[117] Okabayashi S, Uchida K, Nakayama H (2011). Periventricular leukomalacia (PVL)-like lesions in two neonatal cynomolgus monkeys (*Macaca fascicularis*). *Journal of Comparative Pathology*.

[118] Broadbelt KG, Rivera KD, Paterson DS (2012). Brainstem deficiency of the 14-3-3 regulator of serotonin synthesis: a proteomics analysis in the sudden infant death syndrome. *Molecular and Cellular Proteomics*.

[119] Schmitz C, Hof PR (2005). Design-based stereology in neuroscience. *Neuroscience*.

[120] Kopeikina KJ, Carlson GA, Pitstick R (2011). Tau accumulation causes mitochondrial distribution deficits in neurons in a mouse model of tauopathy and in human Alzheimer's disease brain. *American Journal of Pathology*.

